# Air pollution exposure and gestational diabetes mellitus among pregnant women in Massachusetts: a cohort study

**DOI:** 10.1186/s12940-016-0121-4

**Published:** 2016-02-24

**Authors:** Abby F. Fleisch, Itai Kloog, Heike Luttmann-Gibson, Diane R. Gold, Emily Oken, Joel D. Schwartz

**Affiliations:** Division of Endocrinology, Boston Children’s Hospital, 300 Longwood Ave., Boston, MA 02115 USA; Department of Geography and Environmental Development, Ben-Gurion University of the Negev, Beer Sheva, Israel; Department of Environmental Health, Harvard School of Public Health, Boston, MA USA; Channing Laboratory, Brigham and Women’s Hospital, Boston, MA USA; Obesity Prevention Program, Department of Population Medicine, Harvard Medical School and Harvard Pilgrim Health Care Institute, Boston, MA USA; Department of Nutrition, Harvard School of Public Health, Boston, MA USA

**Keywords:** Air pollution, Gestational diabetes, PM_2.5_, Pregnancy

## Abstract

**Background:**

Rodent and human studies suggest an association between air pollution exposure and type 2 diabetes mellitus, but the extent to which air pollution is associated with gestational diabetes mellitus (GDM) is less clear.

**Methods:**

We used the Massachusetts Registry of Vital Records to study primiparous women pregnant from 2003-2008 without pre-existing diabetes. We used satellite-based spatiotemporal models to estimate first and second trimester residential particulate (PM_2.5_) exposure and geographic information systems to estimate neighborhood traffic density. We obtained GDM status from birth records. We performed logistic regression analyses adjusted for sociodemographics on the full cohort and after stratification by maternal age and smoking habits.

**Results:**

Of 159,373 women, 5,381 (3.4 %) developed GDM. Residential PM_2.5_ exposure ranged 1.3–19.3 μg/m^3^ over the second trimester. None of the exposures were associated with GDM in the full cohort [e.g. OR 0.99 (95 % CI: 0.95, 1.03) for each interquartile range (IQR) increment in second trimester PM_2.5_]. There were also no consistent associations after stratification by smoking habits. When the cohort was stratified by maternal age, women less than 20 years had 1.36 higher odds of GDM (95 % CI: 1.08, 1.70) for each IQR increment in second trimester PM_2.5_ exposure.

**Conclusions:**

Although we found no evidence of an association between air pollution exposure and GDM among all women in our study, greater exposure to PM_2.5_ during the second trimester was associated with GDM in the youngest age stratum.

**Electronic supplementary material:**

The online version of this article (doi:10.1186/s12940-016-0121-4) contains supplementary material, which is available to authorized users.

## Background

Gestational diabetes mellitus (GDM) complicates 2–6 % of pregnancies worldwide and as many as 10–20 % of pregnancies in high-risk populations [1]. GDM increases risk of adverse perinatal outcomes such as fetal hypoglycemia and birth trauma to mother and infant. GDM additionally primes infants and mothers for higher risk of cardiometabolic disease later in life [2]. Maternal characteristics such as obesity, older age, and family history of type 2 diabetes mellitus are known to increase risk of GDM, but up to half of women with GDM do not have these classic determinants [3], suggesting a role for environmental factors. Identification of environmental triggers is critical to target at-risk women and provide opportunities for prevention.

Air pollution is one remediable environmental trigger that may predispose susceptible pregnant women to GDM. Automobiles and power plants emit both gasses and particulate air pollutants. The smallest of these particules, with an aerodynamic diameter less than 2.5 μm (PM_2.5_), are readily inhaled. Experimental PM_2.5_ exposure increases insulin resistance in rodents [4, 5] through endothelial dysfunction, inflammation, and oxidative stress [6]. Consistent with the rodent findings, several epidemiologic studies have demonstrated an association between higher air pollution exposure and increased risk of type 2 diabetes mellitus [reviewed in [7]].

However, epidemiologic analyses of prenatal air pollution exposure and abnormal glucose tolerance in pregnancy are conflicting with most [8–11] but not all [12] showing an association. Two of the prior studies were limited by use of birth cohorts with relatively small sample sizes and few cases of GDM [8, 12] and another did not include individual-level socioeconomic status covariate data [9].

In the present analysis, our objective was to use the Massachusetts Registry of Vital Records to evaluate the extent to which first and second trimester residential exposure to PM_2.5_ and neighborhood traffic density were associated with GDM in a large cohort of pregnant women. We hypothesized that prenatal air pollution exposure would be associated with GDM.

## Methods

### Study population and design

**Fig. 1:**
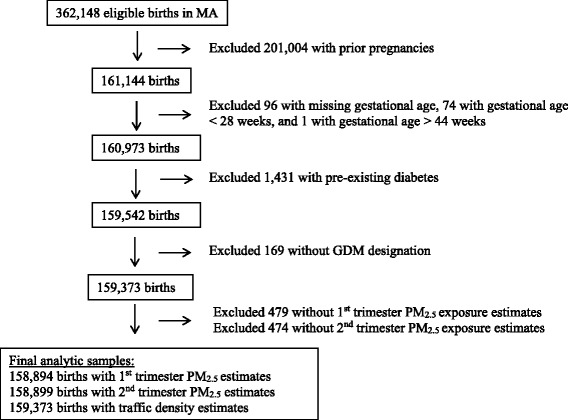
Study flow. Of 362,148 eligible births in Massachusetts from 2003-2008, we included 158,894–159,373 births in final analytic samples. Trimester-specific PM_2.5_ exposure estimates were missing in 0.3 % of cases due to missing daily estimates when data from satellite and/or monitoring stations were not available

We obtained data on registered live births in Massachusetts from January 1, 2003 through December 31, 2008 and latitude and longitude of each residential address at the time of delivery from the Massachusetts Registry of Vital Records and Statistics (http://www.mass.gov/eohhs/gov/departments/dph/programs/admin/dmoa/vitals/). We obtained data only for births associated with a Massachusetts residential address. Daily PM_2.5_ exposure estimates were available as of January 1, 2003, so we included mothers in this analysis whose last menstrual period (LMP) occurred on or after January 1, 2003, which enabled us to create the early pregnancy PM_2.5_ exposure estimates detailed below. Of the 362,148 women who met these inclusion criteria, we restricted the dataset to 161,144 nulliparous women to ascertain only independent observations in our analysis, as the Massachusetts Registry of Vital Records provides a unique ID for each birth rather than each woman. We further restricted our analysis to mothers who delivered at greater than 28 weeks gestation and thus had opportunity for GDM screening, and who did not have a prior history of diabetes. Our final sample included 159,373 women (Fig. 1). The study was approved by the Massachusetts Department of Public Health and the Institutional Review Boards of the participating institutions.

### Exposure assessment

We used each woman’s residential address at the time of delivery to estimate daily PM_2.5_ exposure throughout the pregnancy. To create PM_2.5_ exposure estimates, we used a hybrid satellite-based spatiotemporal model developed by our group, which has been previously described in detail [13]. This model incorporates aerosol optical depth data from the MODIS Satellite and classic land use regression techniques to generate daily PM_2.5_ exposure estimates at the resolution of a 1x1km spatial grid. To create residential exposure estimates, each residential address was linked to the grid in which it was located. The mean daily “out-of-sample” ten-fold cross-validation of the model was excellent (R^2^ = 0.88).

To obtain first trimester exposure estimates, we averaged daily exposures from the date of LMP through 12th week of gestation. To obtain second trimester exposure estimates, we averaged daily exposures from the 13th through 24th week of gestation. To create these time windows, we used the birth certificate clinical estimate of gestational age rather than the estimate calculated based on reported LMP because the former has been shown to be a more accurate predictor of gestational age in birth registries [14], and its use is recommended by the American College of Obstetrics and Gynecology for public health research studies [15].

Our dataset included an estimate of residential traffic density [annual average daily traffic (vehicles/day) times length of road (km) within 100 m of the participants’ residential address at the time of birth] from the Massachusetts Executive Office of Transportation 2002 road inventory, which we used for consistency with prior analyses of this cohort [16, 17]. While our daily estimates of PM_2.5_ were temporally and spatially resolved, estimates of neighborhood traffic density were only spatially resolved.

### Outcome assessment

Routine clinical screening for GDM is recommended for pregnant women in Massachusetts at the end of the second trimester of gestation (24–28 weeks). If serum glucose one hour after a non-fasting 50 g oral glucose challenge test (GCT) is ≥ 140 mg/dL, women are referred for a 3-hour fasting 100-g oral glucose tolerance test (OGTT). As per American Diabetes Association (ADA) criteria, pregnant women are classified as having GDM if they have ≥ 2 of the following abnormal values on the OGTT: blood glucose > 95 mg/dL at baseline, > 180 mg/dL at 1 h, > 155 mg/dL at 2 h, or > 140 mg/dL at 3 h [18]. For this analysis, we obtained maternal GDM designation from the birth records.

### Assessment of covariates

We also retrieved data on other maternal characteristics (age, race/ethnicity, education, smoking status, prenatal insurance, Kotelchuck prenatal care index) as well as infant sex and date of birth directly from birth records. For consistency with prior analyses of this cohort [16, 17], we used data from the 2000 United States Census [19] to calculate median annual household income, percent open space, and median value of owner-occupied housing for the census tract associated with each residential address at the time of birth. We abstracted median annual household income and median value of owner-occupied housing directly from the Census, and we calculated percent open space by intersecting 2000 Census tract boundaries with information on land use for recreation and conservation from MassGIS [20].

### Statistical analysis

We used logistic regression analyses to evaluate the associations of PM_2.5_ exposure and traffic density with GDM. We considered each exposure (first trimester PM_2.5_, second trimester PM_2.5_, and traffic density) in separate models. We first modeled exposures in quartiles to assess for potential non-linearity of exposure-outcome relationships. As we did not see any clear evidence of non-linearity, we also modeled each exposure as a continuous measure and expressed associations per 10–90 percentile increment in exposure. We first fit unadjusted models, followed by full multivariable models for each of the exposures. We included the following covariates potentially associated with air pollution exposure [21, 22] and/or GDM [1, 23]: maternal age (<20 years, 20–35 years, ≥ 35 years), race/ethnicity (white, black, Asian/Pacific Islander, Hispanic, other), education (less than high school, high school, some college, bachelor degree, postgraduate degree), smoking habits [never, former, current low (<10 cigarettes/day), current high (>10 cigarettes per day)], and prenatal insurance (public versus private); census tract median household income (continuous), percent open space (continuous), and median value of owner occupied housing (continuous); season of birth [continuous sine and cosine of date as in [24]]; and date of birth (continuous). Additional adjustment for infant sex and Kotelchuck prenatal care index [25] did not change associations; thus, we did not include these variables in our final models.

We also examined associations between air pollution and GDM within strata of maternal age (<20 years, 20–35 years, ≥ 35 years) in light of data suggesting that children and young adults have higher susceptibility to air pollution [e.g. in relation to respiratory outcomes [26]], presumably due to higher respiratory minute volume, activity level, and time spent outdoors [27]. Within strata of maternal age, we adjusted for maternal age as a continuous variable. In addition, due to evidence for a synergistic interaction between air pollution and smoking in relation to outcomes such as respiratory health [28, 29] and obesity [30, 31], we also examined associations between air pollution and GDM within strata of maternal smoking habits [never, former, current low (<10 cigarettes/day), current high (>10 cigarettes per day)].

We also performed several sensitivity analyses. First, we restricted the sample to women who delivered singleton infants (*n* = 155,501) for consistency with prior studies that examined the relationship between air pollution and GDM among singleton pregnancies only [8–11]. Next, as age appeared to be a strong and nonlinear negative confounder in this population, with older women living in less polluted areas but having a substantially increased risk of GDM (Table 1), we represented age as a cubic (age, age^2^, age^3^) rather than a categorical term in the multivariable model to maximize adjustment for this covariate. In addition, because restricting to nulliparous women decreased our sample size so substantially, we included both nulliparous and multiparous women (*n* = 358,053) in multivariable analyses adjusted for parity in addition to the covariates listed above, with the knowledge that this larger dataset included non-independent observations. Finally, to further investigate the association we identified between second trimester PM_2.5_ exposure and GDM in the youngest mothers, we compared the exposures and sociodemographics of young mothers with versus without GDM. All analyses were conducted using SAS Version 9.4 (SAS Institute Inc, Cary, NC).

**Table 1:** Characteristics of Massachusetts mothers, (1) overall, (2) by quartile of second trimester PM_2.5_ exposure, and (3) in the subset of those with gestational diabetes mellitus (GDM)

		Second trimester PM_2.5_^a^				
Characteristic	Total *n* = 159,373	Q1 *n* = 39,724	Q2 *n* = 39,725	Q3 *n* = 39,725	Q4 *n* = 39,725	With GDM *n* = 5,381
Percent						
Age						
< 20 years	9	8	9	10	11	3
20–35 years	74	74	74	73	74	70
≥ 35 years	17	18	17	17	15	27
Race/ethnicity^b^						
White	70	76	71	68	64	64
Black	7	5	7	8	9	7
Asian/Pacific Islander	8	7	8	9	9	16
Hispanic	12	9	12	13	15	10
Other	2	2	2	3	3	2
Education^c^						
Less than high school	11	9	10	11	13	7
High school	24	22	24	24	25	24
Some college	20	21	20	20	20	24
Bachelor degree	28	30	28	27	26	28
Postgraduate degree	17	18	17	17	17	16
Public prenatal insurance^d^	31	28	31	31	35	26
Smoking habits^e^						
Never	85	84	85	85	86	86
Former	9	10	9	9	8	9
Current low (<10 cigs)	5	5	5	4	4	3
Current high (>10 cigs)	2	2	2	2	2	2
Gestational diabetes	3	4	3	3	3	100
Mean (SD)						
Open space in census tract (%)	12 (11)	13 (11)	12 (11)	11 (11)	10 (11)	11 (11)
Median household income in census tract ($)	51,000 (20,000)	55,000 (19,000)	52,000 (20,000)	51,000 (20,000)	47,000 (19,000)	50,000 (19,000)
Median value of owner occupied housing in census tract ($)	198,000 (120,000)	195,000 (101,000)	193,000 (106,000)	198,000 (116,000)	205,000 (150,000)	184,000 (99,000)

^a^Second trimester PM_2.5_ exposure quartiles mean (SD): Q1 8.2 (0.8), Q2 9.8 (0.4), Q3 11.0 (0.3), Q4 12.6 (0.8). ^b^84 missing from total cohort; ^c^141 missing from total cohort; ^d^42 missing from total cohort; ^e^154 missing from total cohort

## Results

### Population characteristics

Of the 159,373 women in the study population, 5,381 (3.4 %) had GDM. Mean (SD) maternal age at delivery among these primiparous mothers was 28.4 (6.3) years. 70 % of women were white, 31 % had public prenatal insurance, and 85 % were nonsmokers (Table 1).

Mean (SD, range) PM_2.5_ exposure was 10.4 μg/m^3^ (1.7, 3.1–17.1) for the first trimester and 10.4 μg/m^3^ (1.7, 1.3–19.3) for the second trimester. For context, the Environmental Protection Agency (EPA) standard for annual PM_2.5_ exposure was 15 μg/m^3^ during the years of the study. Neighborhood traffic density mean (SD, range) was 1,317 (2,026, 0–37,306) vehicles/day x km of road within 100 m of residential address. Exposures were weakly correlated (Spearman correlation coefficients −0.1 for first with second trimester PM_2.5_, and 0.2 for traffic density with PM_2.5_ in either trimester) (Table 2), consistent with the fact that PM_2.5_ emissions in Massachusetts are primarily from regional rather than local traffic sources [32].

**Table 2:** Distributions of participant air pollution exposure data and correlations between exposures (Spearman r)

	First trimester PM_2.5_ (μg/m^3^)	Second trimester PM_2.5_ (μg/m^3^)	Traffic density^a^
Mean (SD)	10.4 (1.7)	10.4 (1.7)	1,317 (2,025)
Minimum	3.1	1.3	0
Maximum	17.1	19.3	37,306
Spearman r			
First trimester PM_2.5_	1.0	-0.10	0.21
Second trimester PM_2.5_	-0.10	1.0	0.20
Traffic density	0.21	0.20	1.0

^a^Vehicles/day x km of road within 100 m of residential address

Mothers with higher residential PM_2.5_ exposure during the second trimester were more likely to be younger, less educated, and nonsmokers. They were also more likely to have public prenatal insurance and live in a census tract with a lower median household income, less open space, and higher median value of owner-occupied housing (Table 1). Women with GDM (vs. those without) were more likely to be Asian/Pacific Islander. They were also more likely to be older and have private prenatal insurance but live in a census tract with lower median household income, less open space, and lower median value of owner-occupied housing (Table 1). The distribution of characteristics with first trimester air pollution exposure was similar.

### Primary analyses

Residential PM_2.5_ exposure during the first and second trimesters and neighborhood traffic density were not associated with higher odds of GDM in unadjusted or covariate-adjusted analyses. Unadjusted odds of GDM for women in the highest (Q4) (vs. lowest (Q1)) quartile of exposure was 0.92 (95 % CI: 0.85, 1.00) for first trimester PM_2.5_ exposure, 0.98 (95 % CI: 0.90, 1.05) for second trimester PM_2.5_ exposure, and 1.01 (95 % CI: 0.93, 1.09) for neighborhood traffic density. Results of adjusted models were similar [e.g. odds of GDM for Q4 vs. Q1 was 1.00 (95 % CI: 0.96, 1.04) for first trimester PM_2.5_, 0.99 (95 % CI: 0.91, 1.08) for second trimester PM_2.5_, and 1.03 (95 % CI: 0.95, 1.12) for traffic density]. When we represented PM_2.5_ and traffic density exposures as continuous variables (per 10–90 percentile range), relationships with GDM remained null (Table 3).

**Table 3:** Unadjusted and covariate-adjusted^a^ odds ratios (95 % confidence intervals) for gestational diabetes mellitus versus normal glucose tolerance

Exposure	Unadjusted^b^	Covariate-adjusted^c^
First trimester PM_2.5_		
Q1 (3.1–9.3 μg/m^3^)	1.00 (reference)	1.00 (reference)
Q2 (9.3–10.4 μg/m^3^)	0.95 (0.88, 1.03)	1.00 (0.93, 1.09)
Q3 (10.4–11.5 μg/m^3^)	0.91 (0.84, 0.98)	0.97 (0.89, 1.05)
Q4 (11.5–17.1 μg/m^3^)	0.92 (0.85, 1.00)	1.00 (0.92, 1.09)
10–90 percentile range (4.3 μg/m^3^)	0.92 (0.86, 0.99)	1.01 (0.93, 1.09)
Second trimester PM_2.5_		
Q1 (1.3–9.2 μg/m^3^)	1.00 (reference)	1.00 (reference)
Q2 (9.2–10.4 μg/m^3^)	0.98 (0.91, 1.06)	1.04 (0.96, 1.13)
Q3 (10.4–11.6 μg/m^3^)	0.90 (0.83, 0.97)	0.95 (0.88, 1.03)
Q4 (11.6–19.3 μg/m^3^)	0.98 (0.9, 1.05)	0.99 (0.91, 1.08)
10–90 percentile range (4.5 μg/m^3^)	0.96 (0.89, 1.03)	0.97 (0.90, 1.05)
Traffic density^d^		
Q1 (0–280)	1.00 (reference)	1.00 (reference)
Q2 (280–744)	1.02 (0.95, 1.10)	1.04 (0.96, 1.12)
Q3 (744–1,636)	0.99 (0.91, 1.07)	1.01 (0.93, 1.10)
Q4 (1,636–37,306)	1.01 (0.93, 1.09)	1.03 (0.95, 1.12)
10–90 percentile range (2,799)	1.00 (0.96, 1.04)	1.00 (0.97, 1.04)

^a^Adjusted for maternal characteristics (age, race/ethnicity, education, prenatal insurance, smoking habits), census tract characteristics (median household income, percent open space, and median value of owner occupied housing), and timing of birth (season and date)

^b^Sample sizes for unadjusted analyses were 158,894 for associations of first trimester PM_2.5_, 158,899 for second trimester PM_2.5_, and 159, 373 for traffic density

^c^Sample sizes for covariate-adjusted analyses were 158,613 for associations of first trimester PM_2.5_, 158,618 for second trimester PM_2.5_, and 159,025 for traffic density

^d^Vehicles/day x km of road within 100 m of residential address

### Stratified analyses

When we examined the association between air pollution exposure and GDM within strata of maternal age, we observed an association between second trimester residential PM_2.5_ exposure and GDM in mothers who were less than 20 years of age at the time of delivery (Table 4). Within this group of women, those who lived at a residence in the highest (Q4) versus lowest (Q1) quartile of PM_2.5_ exposure during the second trimester had 1.97 (95 % CI: 1.17, 3.32) times the odds of developing GDM. Within this stratum, odds of GDM was consistently higher in Q2, Q3, and Q4 versus Q1 of second trimester PM_2.5_ exposure, and for each 10–90 percentile increment in exposure, odds of GDM was 1.76 times higher (95 % CI: 1.16, 2.7). In mothers greater than 35 years of age at the time of delivery, those in the highest versus lowest quartile of PM_2.5_ exposure during the first trimester had 1.18 (95 % CI: 1.00, 1.39) times the odds of developing GDM, although Q2, Q3, and Q4 versus Q1 odds ratios did not increase monotonically, and, in fact, the Q3 versus Q1 comparison was close to 1. There were no other associations between prenatal pollution and GDM within the age strata (Table 4).

**Table 4:** Covariate-adjusted^a^ odds ratios (95 % confidence intervals) for gestational diabetes mellitus versus normal glucose tolerance, by maternal age. Estimates with 95 % confidence intervals that do not cross the null are bolded

Exposure	<20 years^b^	20- < 35 years^c^	≥35 years^d^
First trimester PM_2.5_			
Q1 (3.1–9.3 μg/m^3^)	1.00 (reference)	1.00 (reference)	1.00 (reference)
Q2 (9.3–10.4 μg/m^3^)	0.84 (0.55, 1.29)	0.98 (0.89, 1.08)	1.08 (0.93, 1.26)
Q3 (10.4–11.5 μg/m^3^)	0.81 (0.52, 1.26)	0.96 (0.87, 1.06)	0.99 (0.85, 1.17)
Q4 (11.5–17.1 μg/m^3^)	0.72 (0.46, 1.13)	0.94 (0.85, 1.04)	1.18 (1.00, 1.39)
10–90%ile range (4.3 μg/m^3^)	0.78 (0.51, 1.88)	0.95 (0.87, 1.04)	1.16 (1.00, 1.35)
Second trimester PM_2.5_			
Q1 (1.3–9.2 μg/m^3^)	1.00 (reference)	1.00 (reference)	1.00 (reference)
Q2 (9.2–10.4 μg/m^3^)	2.12 (1.27, 3.53)	1.03 (0.94, 1.13)	1.00 (0.86, 1.16)
Q3 (10.4–11.6 μg/m^3^)	2.00 (1.19, 3.36)	0.95 (0.86, 1.05)	0.89 (0.76, 1.04)
Q4 (11.6–19.3 μg/m^3^)	1.97 (1.17, 3.32)	1.01 (0.91, 1.11)	0.88 (0.75, 1.04)
10–90%ile range (4.5 μg/m^3^)	1.76 (1.16, 2.69)	0.99 (0.90, 1.08)	0.88 (0.76, 1.03)
Traffic density^e^			
Q1 (0–280)	1.00 (reference)	1.00 (reference)	1.00 (reference)
Q2 (280–744)	1.34 (0.83, 2.17)	1.02 (0.92, 1.12)	1.07 (0.92, 1.24)
Q3 (744–1,636)	1.12 (0.68, 1.84)	1.01 (0.91, 1.11)	1.02 (0.87, 1.19)
Q4 (1,636–37,306)	0.92 (0.55, 1.56)	1.04 (0.95, 1.15)	1.01 (0.86, 1.19)
10–90%ile range (2,799)	0.80 (0.58, 1.11)	1.01 (0.97, 1.06)	0.99 (0.92, 1.06)

^a^Adjusted for maternal characteristics (age, race/ethnicity, education, prenatal insurance, smoking habits), census tract characteristics (median household income, percent open space, and median value of owner occupied housing), and timing of birth (season and date)

^b^Sample sizes for analyses of women < 20 years of age were 14,928 for associations of first trimester PM_2.5_, 14,929 for second trimester PM_2.5_, and 14,974 for traffic density

^c^Sample sizes for analyses of women 20- < 35 years of age were 117,029 for associations of first trimester PM_2.5_, 117,031 for second trimester PM_2.5_, and 117,333 for traffic density

^d^Sample sizes for analyses of women > 35 years of age were 26,656 for associations of first trimester PM_2.5_, 26,658 for second trimester PM_2.5_, and 26,718 for traffic density

^e^Vehicles/day x km of road within 100 m of residential address

Residential PM_2.5_ exposure and traffic density were not consistently associated with GDM within strata of maternal smoking habits. For example, for each 10–90 percentile increment in second trimester PM_2.5_ exposure, odds of GDM was 0.97 (95 % CI: 0.89, 1.05) for women who never smoked, 0.90 (95 % CI: 0.70, 1.16) for women who smoked prior to pregnancy, 1.37 (95 % CI: 0.91, 2.06) for women who smoked less than 10 cigarettes per day, and 0.98 (95 % CI: 0.52, 1.83) for women who smoked more than 10 cigarettes per day (data not shown).

### Sensitivity analyses

When we restricted the sample to women with singleton pregnancies and when we represented age as a cubic rather than a categorical term, there was no change to the pattern of results (data not shown). When we included multiparous women, there was no change to the pattern of results as compared to our primary analyses with the exception of the following: (1) we found no association between first trimester PM_2.5_ and GDM in mothers greater than 35 years of age, and (2) odds of GDM was higher per 10–90 percentile range increment of first and second trimester PM_2.5_ exposure in the subset of mothers who smoked less than 10 cigarettes per day during pregnancy [OR: 1.34 (95 % CI: 1.06,1.68) for first trimester PM_2.5_ and 1.30 (95 % CI: 1.03,1.65) for second trimester PM_2.5_] (data not shown). When we compared young mothers with versus without GDM, sociodemographics were generally similar between the groups, although those with GDM were less likely to smoke, more likely to have attended some college, and more likely to live in census tracts with somewhat lower median household income, less open space, and lower median value of owner-occupied housing. Young mothers with versus without GDM had higher residential PM_2.5_ exposure during the second trimester, but lower neighborhood traffic density (Additional file 1).

## Discussion

In our analysis of Massachusetts birth registry data from 2003-2008, pregnant women with high residential PM_2.5_ exposure during the first or second trimester or high neighborhood traffic density had the same odds of developing GDM as women with lower exposures. When we examined this association within strata of maternal age, the youngest mothers (<20 years of age) had increased odds of GDM when exposed to higher residential PM_2.5_ during the second trimester.

Our findings are consistent with prior population-based studies that have shown an association between GDM and prenatal exposure to NO_x_ or ozone [9–11] but found weaker or no associations with exposure to PM_2.5_ [8, 10, 11] or traffic density [8, 9, 12]. The overall lack of a consistent association between PM_2.5_ exposure and GDM in human observational studies is in contrast to a growing body of epidemiologic literature showing an association between PM_2.5_ and type 2 diabetes mellitus, [reviewed in [7]] and rodent studies confirming an association between PM_2.5_ and insulin resistance in non-pregnant adults [4, 33]. PM_2.5_ is primarily thought to lead to insulin resistance through oxidative damage, endothelial dysfunction, and inflammation [4, 34], whereas the specific mechanisms of NO_x_ and ozone-induced insulin resistance are not as well-understood. In one rodent study, ozone-induced insulin resistance was associated with neuronal activation and sympathetic stimulation [35] but not with the increase in circulating inflammatory cytokines that has been observed following PM_2.5_ exposure [4, 33]. Pregnant women may be more vulnerable to NO_x_ or ozone-specific mechanisms, and the role of each of these pollutants and mechanisms of action in relation to GDM should be studied in pregnant rodent models.

Although prenatal residential PM_2.5_ exposure and traffic density were not associated with GDM in analyses of our complete study population, we found higher second trimester PM_2.5_ exposure (i.e.— quartiles 2–4 vs. the lowest quartile) to be associated with almost twice the odds of GDM in the subset of women less than 20 years of age. Second trimester exposures are biologically relevant, as GDM is a pathologic exacerbation of a physiologic increase in insulin resistance that occurs specifically during the second trimester of pregnancy [36]. Also, the magnitude of our finding is similar to that of other well-known risk factors for GDM. For example, in a meta-analysis, overweight (vs. normal weight) mothers had 1.83 (95 % CI: 1.58, 2.12) times the odds of developing GDM, and those who were obese had 3.52 (95 % CI: 3.24, 3.84) times the odds [37]. Our finding of an association between air pollution and GDM in the youngest mothers may be because maternal age is such a strong risk factor for GDM [1] that older women destined to develop GDM will do so regardless of other factors, whereas young women are more readily influenced by environmental exposures such as air pollution. Additionally, as compared to older adults, children and young adults have been shown to be more susceptible to PM_2.5_-induced health outcomes [26], presumably as a result of higher respiratory minute volume, activity level, and time spent outdoors [27].

We also considered alternative explanations for our finding of an association between PM_2.5_ exposure and GDM in the youngest mothers. For example, the possibility that stratification could have reduced negative confounding by age is less likely because PM_2.5_ was not associated with GDM in the other age strata, and the association in the full cohort was null even when we included age as a cubic term. Alternatively, our inability to account for pre-pregnancy BMI, which was not recorded on Massachusetts birth certificates until 2011, could have confounded the association in the youngest mothers. However, if there were counfounding by pre-pregnancy BMI, we would have expected this to have affected all mothers equally, rather than observing it in the youngest age strata only, based on a prior analysis of >170,000 women in which the association between BMI and GDM did not vary by maternal age [38]. Another possible explanation is differential composition of residential PM_2.5_ exposure by maternal age (e.g. higher traffic-related and/or ultrafine particle component of PM_2.5_ most prevalent in the lower SES neighborhoods of the youngest mothers and also most closely related to GDM). However, there was no association between traffic density and GDM in our complete study population, and we found lower rather than higher neighborhood traffic density in the subset of young women with versus without GDM. It is possible that our finding of an association between PM_2.5_ and GDM in the youngest mothers may also reflect random chance, particularly given the relatively small number of cases of GDM (*n* = 179) in the youngest age stratum. Our findings require replication in other populations of young, pregnant women.

In our sensitivity analysis which included multiparous women, first and second trimester PM_2.5_ exposure was associated with slightly increased odds of GDM in the subgroup who smoked less than 10 cigarettes per day during pregnancy. Tobacco smoke contains particulates and has previously been shown to operate synergistically with air pollution to increase risk of obesity, another cardiometabolic outcome [30, 31]. However, the fact that this association was not present in the heavier smokers (greater than 10 cigarettes per day) makes it difficult to draw a definitive conclusion and suggests the need for replication in other cohorts.

Use of data from the Massachusetts Registry of Vital Records is a unique strength of our study. The registry contains pregnancy data on all Massachusetts residents, includes a large number of cases of GDM, and is free from the selection bias typical of cohort studies. The registry also contains relatively rich data on socioeconomic status, as well as smoking and diabetes history. Also, our PM_2.5_ model, which leveraged satellite aerosol optical depth data, calculated estimates at a fine resolution and had a high mean out-of-sample *R*^2^.

Potential exposure and outcome misclassification are limitations of the present study that may have biased results toward the null. We did not have information on time-activity patterns or residential moves during pregnancy which could have improved accuracy of exposure estimates. Also, traffic density estimates were for 2002 which may not have been relevant for the whole study period. Outcome misclassification could have occurred as a result of underreporting of GDM on the birth certificate (specificity >98 % and sensitivity 46–83 % when compared to medical records) [39] or because women with undiagnosed type 2 diabetes mellitus may have inappropriately been included in the GDM group. In addition, based on limited information available in the birth registry, we were unable to account for every factor that might be related to pollution exposure and GDM risk, such as physical activity, family history of GDM, and maternal pre-pregnancy BMI. Also, census tract covariates utilized 2000 Census tract data which may not have been relevant for the whole study period, and thus residual confounding may exist. Also, we did not have exposure estimates available in this dataset for other criteria pollutants such as NO_x_ or SO_2_. Overall PM_2.5_ exposure was low, and it may be that higher levels of exposure are associated with greater risks.

Additional rodent studies are needed to elucidate the extent to which individual criteria pollutants such as PM_2.5_, NO_x_, SO_2_, black carbon, and ozone, and mixtures of pollutants, are causally linked to development of GDM and to further investigate mechanisms of action. Large population-based studies with information on multiple criteria pollutants and covariates are also needed, particularly in young cohorts with otherwise low risk of GDM.

## Conclusions

Although our findings require replication, in our cohort, young women were at increased risk for GDM when exposed to higher residential PM_2.5_ during the second trimester of pregnancy. We otherwise found no consistent evidence of an association between first or second trimester residential PM_2.5_ exposure or neighborhood traffic density and GDM in pregnant women in Massachusetts with modest levels of air pollution exposure.

## Additional file

Additional file 1:
**Characteristics of Massachusetts mothers (1) overall, (2) < 20 years old with gestational diabetes mellitus (GDM) and (3) < 20 years old without GDM.** (PDF 16 kb)
