# Supplementary material for: Air pollution exposure and gestational diabetes mellitus among pregnant women in Massachusetts: a cohort study
Source: Environ Health. 2016 Feb 24;15:40. doi: 10.1186/s12940-016-0121-4 (PMC4765142; doi:10.1186/s12940-016-0121-4)
Supplement: Additional file 1: — Characteristics of Massachusetts mothers (1) overall, (2) < 20 years old with gestational diabetes mellitus (GDM) and (3) < 20 years old without GDM. (PDF 16 kb) [file 12940_2016_121_MOESM1_ESM.pdf]

Additional file 1. Characteristics of Massachusetts mothers (1) overall, (2) < 20 years old with gestational diabetes mellitus (GDM) and (3) < 20 years old without GDM

**Characteristic**	**Total cohort (n=159,373)**	**Young women with GDM (n=178)**	**Young women without GDM (n=14,820)**
		**Percent**	
**Race/ethnicity**[a]			
White	70	44	46
Black	7	10	12
Asian/Pacific Islander	8	5	4
Hispanic	12	37	35
Other	2	5	3
**Education**[b]			
Less than high school	11	57	58
High school	24	33	37
Some college	20	10	5
Bachelor degree	28	0	0
Postgraduate degree	17	0	0
**Public prenatal insurance**[c]	31	76	74
**Smoking habits**[d]			
Never	85	82	75
Former	9	8	12
Current low (<10 cigs)	5	4	10
Current high (>10 cigs)	2	6	3
		**Mean (SD)**	
**Maternal age (years)**	28.4 (6.3)	18.1 (1.0)	18.0 (1.1)
**Open space in census tract (%)**	12 (11)	9 (9)	9 (10)
**Median household income in census tract ($)**	51,214 (19,595)	37,517 (15,075)	38,044 (15,295)
**Median value of owner occupied housing in census tract ($)**	197,812 (119,745)	130,318 (54,097)	138,232 (66,610)
**1st trimester $PM_{2.5}$ (μg/m^3)**	10.4 (1.7)	10.3 (1.7)	10.5 (1.7)
**2nd trimester $PM_{2.5}$ (μg/m^3)**	10.4 (1.7)	10.9 (1.7)	10.6 (1.8)
**Traffic density**[e]	1,317 (2,026)	1,223 (1,202)	1,411 (1,810)

[a]84 missing from total cohort; [b]141 missing from total cohort; [c]42 missing from total cohort; [d]154 missing from total cohort [e]Vehicles/day x km of road within 100 m of residential address
